# Synergistic effect of TiO_2_ nanoparticles and poly (ethylene-co-vinyl acetate) on the morphology and crystallization behavior of polylactic acid

**DOI:** 10.1038/s41598-024-68023-4

**Published:** 2024-08-05

**Authors:** Safaa H. El-Taweel

**Affiliations:** 1 Chemistry Department, Faculty of Science, Cairo University, Orman-Giza, 12613 Egypt; 2 Engineering and Materials Science Department, German University in Cairo, New Cairo City, Egypt

**Keywords:** Polylactic acid (PLA), Poly(ethylene-co-vinyl acetate), With vinyl acetate content 80 (EVA80), DSC, MDSC, Modified Avrami, Effective activation energy, POM, SEM, Chemistry, Biomaterials, Materials science, Polymers

## Abstract

The impact of adding ethylene vinyl acetate copolymer (EVA 80) and 1 wt% TiO_2_ nanoparticles on the morphology and crystallization behavior of poly(lactic acid) blends was investigated using DSC, SEM, and POM. Thermal analysis revealed the enhancement of crystallinity of PLA in the presence of TiO_2_ and higher EVA 80 content in the blend. The PLA and EVA 80 components showed compatibility, as evidenced by the shift of the glass transition temperatures of the PLA phase in the blend to lower values compared to neat PLA. The lower temperature shift of the cold crystallization of the PLA and the formation of the small spherulites of the PLA in the blends indicated that the EVA 80 and TiO_2_ act as a nucleating agent for crystallization. The non-isothermal crystallization parameters of the composites were evaluated using Avrami's modified model, the MO approach, and Friedman’s isoconversional method. The Avrami’s modified rate constant (K) and the effective activation energy values significantly increased with the incorporation of EVA 80 and TiO_2_ nanoparticles. Furthermore, the thermogravimetric analysis (TGA) showed improved thermal stability of PLA by adding EVA 80 and TiO_2_.

## Introduction

Poly(lactic acid) (PLA) is a biodegradable polymer that can be produced by the fermentation of lactic acid derived from renewable resources such as cellulose and starch and was primarily chosen to address environmental issues often associated with non-biodegradable polymers. Because of its beneficial characteristics, including outstanding biodegradability, high tensile strength, and high transparency, polylactic acid (PLA) has recently received much attention^1–6^. Despite the various benefits of PLA, its limited high modulus, low strain, and low thermal stability restrict its widespread use. Adding reinforcing fillers, especially nanofillers, to the PLA matrix is a method used to enhance mechanical and thermal properties, as well as to improve barrier qualities. Nanoparticles as fillers in polymers have gained significant interest for enhancing thermal, mechanical, and barrier properties at low filler concentrations. Titanium dioxide (TiO_2_) nanoparticles are thermally stable, non-toxic, environmentally friendly, and have antimicrobial and UV protection capabilities^7–11^. Several studies showed that adding TiO_2_ nanoparticles into the PLA matrix improves some drawbacks of neat PLA and expands its wide applications^5,9–12^.

On the other hand, the blending of PLA with various elastomers^9,13–21^, including poly(ethylene-co-vinyl acetate) (EVA) copolymers^22–25^, was employed to modify the physico-chemical properties of PLA. EVA is a copolymer comprising two homopolymer components: polyethylene and poly(vinyl acetate) (PVAc). It exhibits qualities that fall between those of the two individual polymers. EVA has been utilized as a biomaterial in artificial heart applications and drug delivery because of its safety and biocompatibility^23^. As the vinyl acetate (VA) concentration increases, EVA copolymers transition from a semicrystalline thermoplastic material (as Low-density polyethylene) to rubber and then to an amorphous thermoplastic material(as polyvinyl acetate)^26^. PLA and poly(vinyl acetate) (PVAc) are known to be miscible^27^ and PLA exhibits phase separation when blended with LDPE^28^. Due to its significant rubber and resin characteristics, EVA copolymer has been mixed with PLA. Adjusting the VA component of EVA copolymer can achieve compatibility with PLA^29–32^ without needing a further compatibilizer. Ma et al.^29^ investigated how the VA content of EVA affected the characteristics of the PLA/EVA blend with (80:20) using compression-molded samples; they discovered that EVA50 is a highly effective PLA toughening agent. There was a significant increase in impact properties and strain at break upon adding EVA to PLA. The two immiscible components have been reported for PLA and EVA, with VA content of 18–70%^29,33^. However, EVA 85 has been reported to be completely miscible with PLA^29,33^.

Sangeetha et al.^34^ examined the influence of the blend ratio of PLA/EVA 40 (95:5, 90:10, 85:15, and 80:20) on the mechanical and thermal properties. The researchers discovered that incorporating 15% EVA40 into the PLA matrix considerably enhanced the impact strength of the PLA/EVA40 blend. Through the investigation of morphology, a correlation was revealed between the rise in EVA content and the increase in the size distribution of EVA40 particles. Additionally, there is an immiscibility between EVA40 and PLA. Agrawal et al.^35^ demonstrated a similar trend for PLA/EVA19.Cong et al.^15^, Aghjeh et al.^18^, combined (EVA18) with PLA. Nevertheless, they could only attain a modest enhancement in ductility (in terms of strain or impact strength) even when the EVA18 content reached 20–30%, primarily because of the absence of compatibility between the different phases. Ta´bi et al.^36^ demonstrated the nucleation capacity of EVA60 content in PLA. Furthermore, when 20% EVA60 was added, the PLA’s crystallinity rose from 20 to 36%. In addition, the impact strength of PLA was enhanced due to the synergistic effect between the annealing of PLA (at 80°, 100°, 120°, and 140 °C) and the presence of EVA60.

To our knowledge, the literature has yet to study the impact of a combination of 1%TiO_2_ and EVA 80 (10%, 30%, and 50%) on the thermal and crystallization behavior of PLA.

Only a few works in literature investigated the effect of EVA80 copolymer on the thermal and crystallization behavior of PLA and its correlation with the morphology and compatibility of PLA/EVA80 blends. TiO_2_ nanoparticles to PLA enhance its mechanical properties, thermal stability, antibacterial and UV resistance. The choice to mix EVA80 and TiO_2_ is to keep the PLA echo-friendly, biodegradable, and safe for applications. Overall, the combination of PLA and EVA 80 and 1 wt% TiO_2_ may offer a versatile and sustainable solution for a wide range of industries, including medical devices and packaging materials.

This work aims to evaluate the synergetic impact of different EVA80 content and 1 wt% TiO_2_ content on PLA’s thermal properties and morphology. Additionally, optimizing the compatibility of polymer blends can result in improved processing characteristics, such as easier melt processing and better adhesion between different polymer phases. Therefore, the compatibility and miscibility of composite components were investigated using scanning electron microscopy (SEM) and modulated DSC. The investigation of PLA/EVA80/1 wt% TiO_2_ composites involved in studying the nonisothermal cold crystallization behavior and kinetics using differential scanning calorimetry (DSC). This is important because most PLA processing techniques occur under nonisothermal conditions, and understanding the connection between processing and properties relies on considering PLA's crystallization kinetics. Avrami's Modified approach was used to analyze experimental data. The activation energy was calculated using the isoconventional Friedmann approach. Polarized optical and scanning electron microscopy were used to examine the morphology and dispersion of EVA 80 and TiO_2_ into the PLA matrix. The influence of thermal stability was investigated by thermal gravimetry analysis (TGA).

## Materials

The PLA, composed of 99% l-lactic acid and 1% d-lactic acid, was obtained from Biomer Company (Krailling, Germany). The commercial grade of PLA is known as Biomer L9000 and has an average molecular weight of 200 kDa with a polydispersity index of 1.98. The PLA was received as white pellets and was used after drying at 70 °C for 3 h. Lanxess Co., Germany, supplied the poly(ethylene-co-vinyl acetate) (EVA80). It comprised 20 mol% ethylene and 80 mol% vinyl acetate (EVA 80). Additionally, 99.9% pure chloroform was purchased from Sigma Aldrich. Titanium chloride (TiCl_4_, 99.90%) and Concentrated Sulfuric acid (98%) were acquired from Fisher. Ammonium hydroxide was acquired in a concentrated form from Sigma-Aldrich. All chemicals were obtained commercially and used without further purification.

### Synthesis of TiO_2_ nanoparticles

**Scheme 1:**
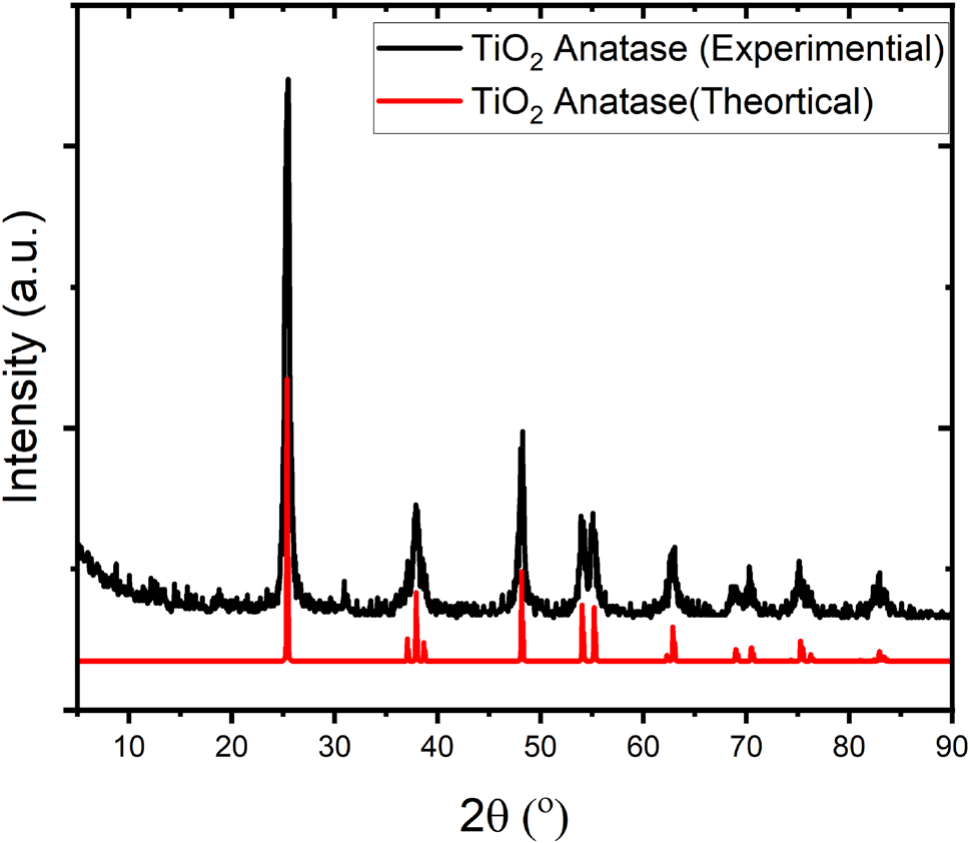
XRD pattern for produced TiO_2_ calcinated at 500 °C.

Titanium dioxide (TiO_2_) nanoparticles with anatase structure were synthesized by hydrolysis of TiCl_4_ in H_2_SO_4_ solution in the ice bath at 5 °C, precipitation with NH_4_OH, and the calcination for 2 h at 500 °C, according to the method described before^8^. In brief, a certain mass of TiCl_4_ was dissolved in distilled water to adjust the concentration to 1.0 M. Then the TiCl_4_ solution was added dropwise into 1.0 M H_2_SO_4_ solution while stirring at an ice bath. A grey solution was produced through ongoing stirring followed by heating to 70 °C for 2 h until it became a clear solution. Then, the reaction mixture was cooled at room temperature, and the concentrated NH4OH solution was dropped wisely to adjust the pH value at 7. The resulting precipitate (hydrated titanium hydroxide) was separated by filtration and washed several times with distilled water until the Cl^-^ from the liquid disappeared. Subsequently, the solid was dried at 110 °C for 24 h in a vacuum oven and then calcined for 2 h at 500 °C. The crystal structure of the calcined sample was examined by X-ray diffraction (Scheme 1). As can be seen from this figure, the sharp, intense diffraction peaks observed at 2θ = 25.41°, 30.98°, 37.92°, 48.30°, 53.97°, 55.09°, 62.82°, 68.91°, 70.30°, 75.06° and 83.03° which are ascribed to (101), (103), (004), (112), (200), (105), (204), (116), (220), (215) reflections, respectively, confirms the anatase structure of TiO_2_. As calculated using Debye–Scherrer's equation^8^ the average size of the crystals was 18.1 nm.

**Scheme 2:**
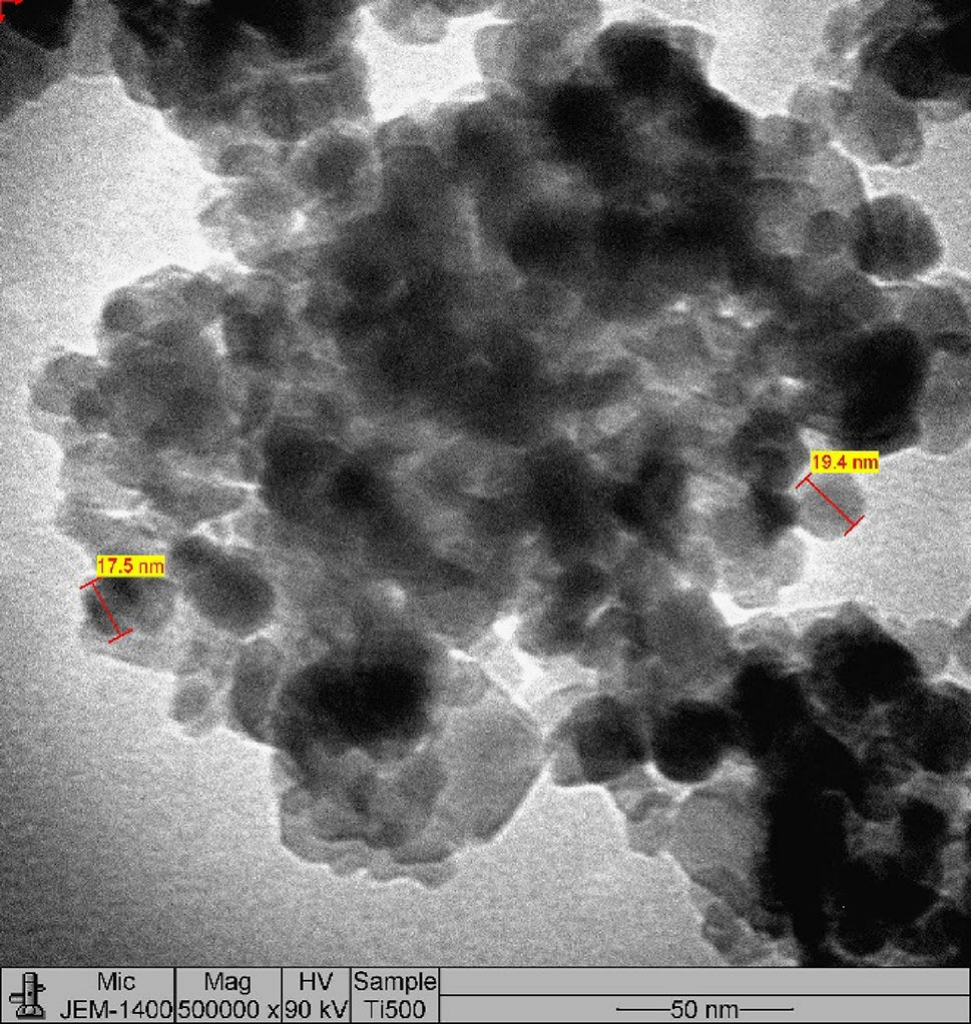
TEM micrograph for TiO_2_ calcinated at 500 °C.

In addition, the TiO_2_ morphology was observed through a TEM image (Scheme 2), which showed that the NPs exhibited a nearly spherical shape with an average diameter of 19.1 nm.

### PLA/EVA 80 composites preparation

PLA/EVA80 blends with compositions (100/0, 90/10, 70/30, and 50/50) containing 1 wt% TiO_2_ nanoparticles were made using the casting film method with chloroform solvent. The mixture was stirred at 50 °C for 24 h and then sonicated for 1.0 h at room temperature to ensure complete dissolution of the PLA and EVA80 and good dispersion of TiO_2_ in the chloroform. Subsequently, the concoction was carefully transferred onto the glass Petri dish and allowed to remain undisturbed at ambient temperature for 7 days, during which the solvent gradually dissipated, yielding a uniform sheet. After that, the cast film was placed on a hot melt press at 200 °C for 2 min and then was cooled to room temperature.

### Characterization

The transition temperatures of the sample were examined using a DSC-Q100, the RCS90 refrigerator cooling system from TA Instruments Co., and Universal Analysis 2000 software. The DSC's temperature and heat flow were calibrated using the suggested technique^37^, and all DSC scans were performed in a nitrogen environment (30 ml/min). The samples were placed in DSC aluminum crucibles. The samples were first heated from 0 to 200 °C at 10 °C/min. This was done to eliminate any previous thermal effects and enhance thermal connection. Next, they were cooled to 0 °C at a cooling rate of 10 °C/min, and finally, they were heated to 200 °C at a rate of 10 °C/min. Data from the first heating run are not discussed. The temperatures of cold crystallization (Tcc) and melting (Tm) were calculated from the maxima of the exothermal and endothermal processes and the corresponding enthalpies (ΔH_CC_ and ΔH_m_) from the second heating scans. The total crystallinity of PLA (Xc) was calculated using Eq. (1).

1 $${X}_{c}=\frac{\Delta {H}_{m}}{\Delta {H}_{m}^{^\circ }\times m}$$

where $\Delta {H}_{m}$ is the melting enthalpy of 100% crystalline PLA, which has been determined from the literature and equals 93.6 J/g^20^, and $m$ represents the mass proportion of PLA in its blend.

For the non-isothermal crystallization study, the samples were kept at 200 °C for 3 min and then rapidly cooled at 60 °C /min until the temperature reached 0 °C. DSC curves were collected for different heating rates (3, 5, 7, and 10 °C/min) to study non-isothermal crystallization behavior. These runs were used to calculate the crystallization temperature (T_CC_) and the enthalpy of crystallization (ΔH_CC_) during the heating run.

The glass transition temperature for PLA: EVA80:1 wt%TiO_2_ was investigated using modulated differential scanning mode. The samples were first annealed at 200 °C for 2 min to remove the previous thermal history. After that, they were cooled to − 60 °C at 60 °C/min and kept for 2 min. Then, the modulated heating was started from − 60 to 100 °C, with an underlying heating rate of 1 °C/min, a period of 60 s, and an amplitude of 1 °C. These parameters allowed us to observe the glass transition as a step change in the reversing heat capacity curve and peak in the heat flow phase curve.

### Polarized optical microscopy

An Imager A1 polarized light optical microscope (Zeiss, Germany) with a digital camera was used to study the crystallization morphology of PLA-based samples. We placed each sample between two thin glass slides and heated it to 200 °C on a hot plate for 3 min. The samples were quickly lowered to 125 °C. Both composites and neat PLA were crystallized at 125 °C. We captured images of polarized optical micrographs after a 90-min annealing process at 125 °C.

### Scanning *electron* microscopy

PLA composites were examined by scanning electron microscopy (SEM) using a JEOL 2000(Japan) apparatus. The surface specimens were viewed after being sputter-coated with a thin layer of gold to prevent electrostatic charges during the examination.

## Results and discussion

**Figure 1:**
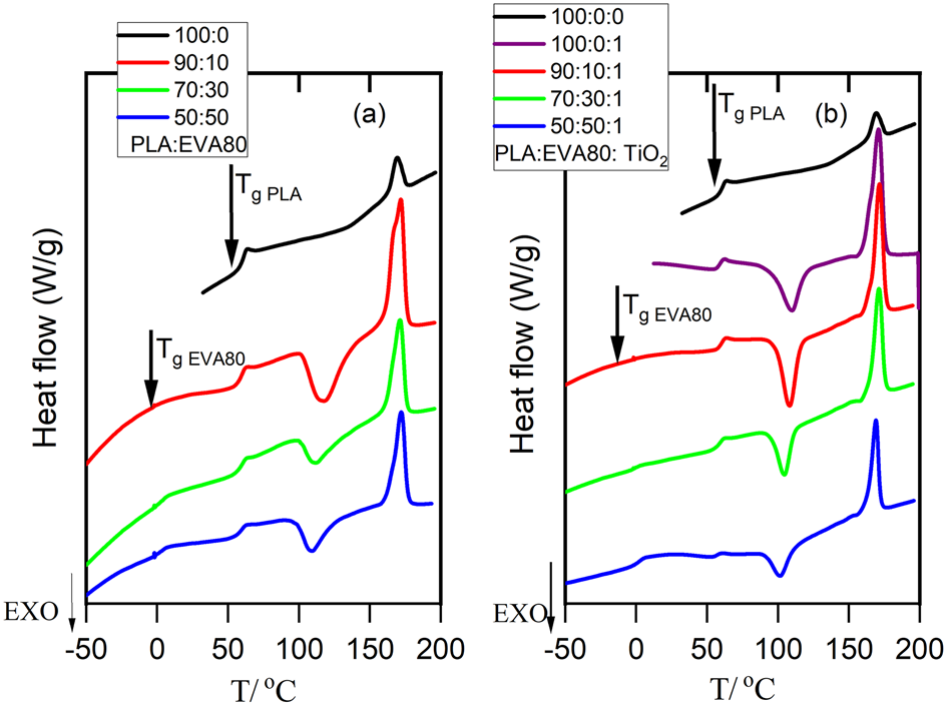
DSC second heating curves for (**a**) PLA: EVA80; (**b**) PLA: EVA80:TiO_2_.

No distinguishable melt crystallization peak was detected for neat PLA, PLA/EVA80, and PLA/ EVA80/1wt% TiO_2_ samples while cooling from a melt even at 10 K min^−1^, indicating that PLA chain crystallization is entirely impeded at this rate. However, a melt crystallization peak was observed for PLA/1 wt% TiO_2_ and occurred at 97.6 °C. These results are consistent with TiO_2_ nanoparticles, which have been reported to be an efficient nucleating agent for PLA^9,11,38^. On subsequent heating, an exothermic cold crystallization peak is detected for PLA-based samples, as shown in Fig. 1. Figure 1-a shows that the cold crystallization peak of PLA became sharper and shifted to a lower value with the incorporation of EVA 80 into the PLA matrix. This can be elucidated by the fact that EVA80 made PLA macromolecular chains more mobile, generating favorable conditions for PLA crystallization, as reported before^15,24,39,40^. Another possible reason is that a heterogeneous structure between PLA and EVA80 accelerates the PLA’s crystallization process, as Tien et al.^41^ mentioned. Figure 1-b shows the DSC second heating run of PLA: EVA80:1 wt% TiO_2_ The inclusion of 1 wt% TiO2 in PLA/EVA 80 caused the cold crystallization peak to shift to a lower temperature, as illustrated in Fig. 1-b and Table 1. Moreover, the cold crystallization peak became sharper, narrower, and stronger than PLAEVA 80, as shown in Fig. 1b. These results indicated that TiO_2_ nanoparticles act as an efficient nucleating agent. A narrow, sharp, single melting peak was observed for PLA: 1 wt% TiO_2_ and PLA: EVA 80: 1 wt% TiO_2_. EVA 80 content and TiO_2_ nanoparticles slightly influenced the maximum melting temperature, as shown in Fig. 1. A similar trend was reported for PLA/nano-bio composites^42^ and PLA/PHO/Talc^6^. The heat of crystallization depends on the thermal history of the polymer. It is also vital to know the percent of crystallinity since it substantially affects the physical properties of the polymer. The degree of crystallinity of PLA, PLA/EVA80, and PLA/EVA80/1 wt% TiO_2_ composites was determined using Eq. (1) and presented in Table 1.

**Table 1:** Thermal parameters of PLA/EV80 and PLA/EVA80/1 wt.% TiO_2_ blends from DSC (cool and second heating) curves.

PLA samples	${T}_{c}$±1	$\Delta {H}_{c}$±5	${T}_{cc}$±1	$\Delta {H}_{cc}$±5	${T}_{m}$±1	$\Delta {H}_{m}$±5	${\rm X}_{c}$
	°C	J/g	°C	J/g	°C	J/g	%
PLA: EVA80							
100:0	nd	nd	136	4	169	9	10
90: 10	nd	nd	118	31	171	45	53
70: 30	nd	nd	113	17	171	31	47
50:50	nd	nd	110	15	169	23	49
PLA:EVA80: TiO_2_							
100:0:1	98	8	110	33	171	47	50
90:10:1	nd	nd	108	30	172	44	52
70:30:1	nd	nd	105	20	171	34	52
50:50:1	nd	nd	101	17	169	28	60

The presence of TiO_2_ and EVA 80 significantly increases the degree of crystallinity of PLA, as shown in Table 1.

**Figure 2:**
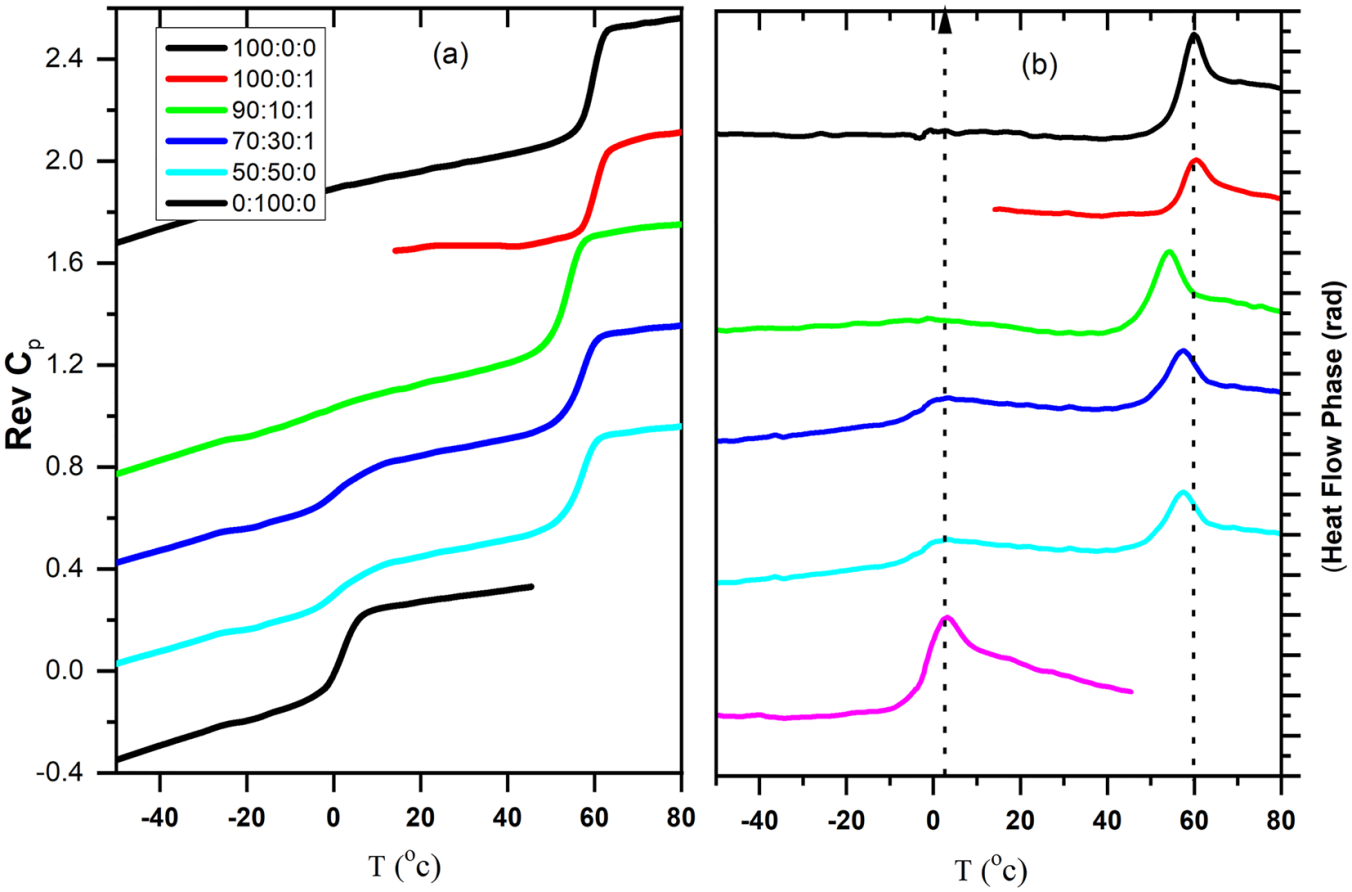
TMDSC curves in the glass transition region of the PLA: EVA80: 1 wt% TiO_2_; (**a**) reversing Cp; (**b**) phase angle.

Modulated DSC runs were conducted to investigate the miscibility between PLA and EVA 80, as shown in Fig. 2. It is well known that glass transition temperature is accompanied by a step change in the ‘reversing heat capacity and a peak in the phase signal. The values of glass transition temperatures of pure PLA and pure EVA 80 are 60 °C and 0 °C, respectively, as shown in Fig. 2 and Table 2. Two glass transition temperatures are observed for PLA: EVA80: 1 wt% TiO_2_. The position of the glass transition temperature of PLA changes slightly and shifts to a lower value (around 4 °C) with the addition of EVA80, indicating the plasticization effect of EVA80. The depression of cold crystallization temperature and a slight lowering of PLA’s modulated glass transition temperature may indicate the strong compatibility of PLA and EVA80. Several authors have reported that the vinyl acetate content influenced the compatibility and phase morphology of the PLA/EVA blends in the random copolymers^25,29,43^. The phase separation between the two immiscible components has been reported for EVA, with VA content of 18–70%. However, EVA with VA content 85 and 90 have been reported to be entirely miscible with PLA^29,33^.

**Table 2:** Glass transition temperature from modulated DSC, Peak is taken from a maximum of heat flow phase (rad).

Sample	T_g EVA_ (°C)	ΔCp_EVA_	T_g PLA_ (°C)	ΔCp_PLA_	Peak_1_	Peak_2_
100:0:0	–	–	60	0.4	–	60
100:0:1			60	0.38	–	60
90:10:1	–	0.04	54	0.42	1.5	54
70:30:1	0.8	0.15	57	0.33	3	57
50:50:1	1.4	0.14	55	0.33	2	55
0:100:0	2	0.31	nd	nd	3	nd

### Scanning *electron* microscopy

**Figure 3:**
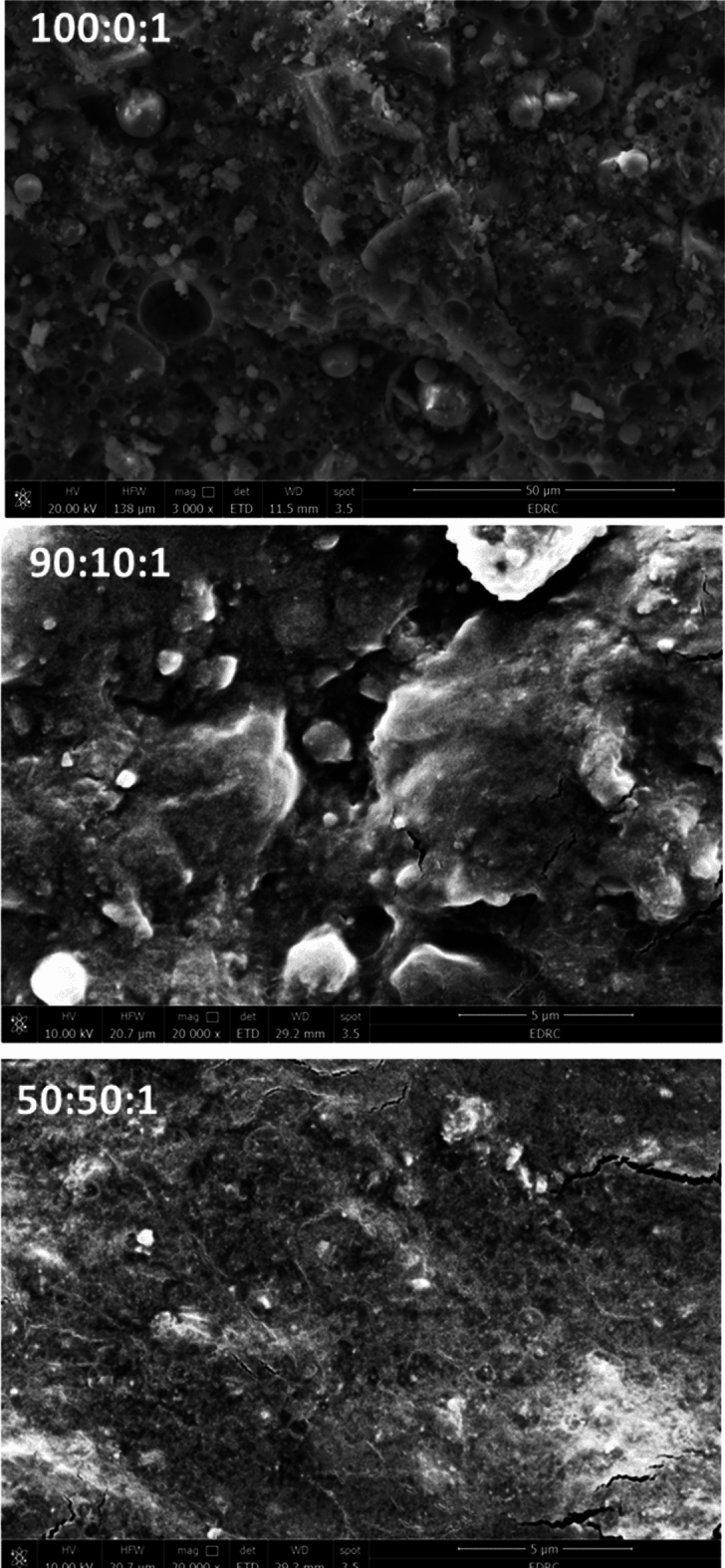
SEM micrographs for PLA: EVA80: 1 wt% TiO_2_.

Figure 3 illustrates SEM images for PLA/1 wt%TiO_2_ and PLA/EVA80/1 wt% TiO_2_.

SEM images reveal that the spherical shape of TiO_2_ nanoparticles is randomly distributed in the PLA matrix. The fracture surface of PLA:TiO_2_ (100:0:1) shows the features of a very brittle material. The phase morphology of a polymer blend is mainly influenced by its miscibility. The roughness of the surface decreases as the EVA 80 content. No discrete domains of EVA 80 and many elongated fibrils were observed, especially for 50 wt% EVA 80. The elongated shape was formed as a result of the soft EVA 80 phase flowing into the PLA hard phase during the sample's fabrication. These results reflect the compatibility of PLA and EVA80 in the blend. It is worth noting that PLA and EVA became thermodynamically miscible when the vinyl acetate content in copolymer was increased to 85 wt%, and no phase separation occurs in compatible blends like PLA/PVAC and PLA/EVA 90 as reported in Refs.^27,33^. The presence of EVA's polar vinyl acetate groups to interact with PLA enough accounts for PLA and EVA's compatibility, as reported in Ref.^43^, which confirms the shift of glass transition temperature of the PLA phase in the blend.

### Polarized optical microscopy

**Figure 4:**
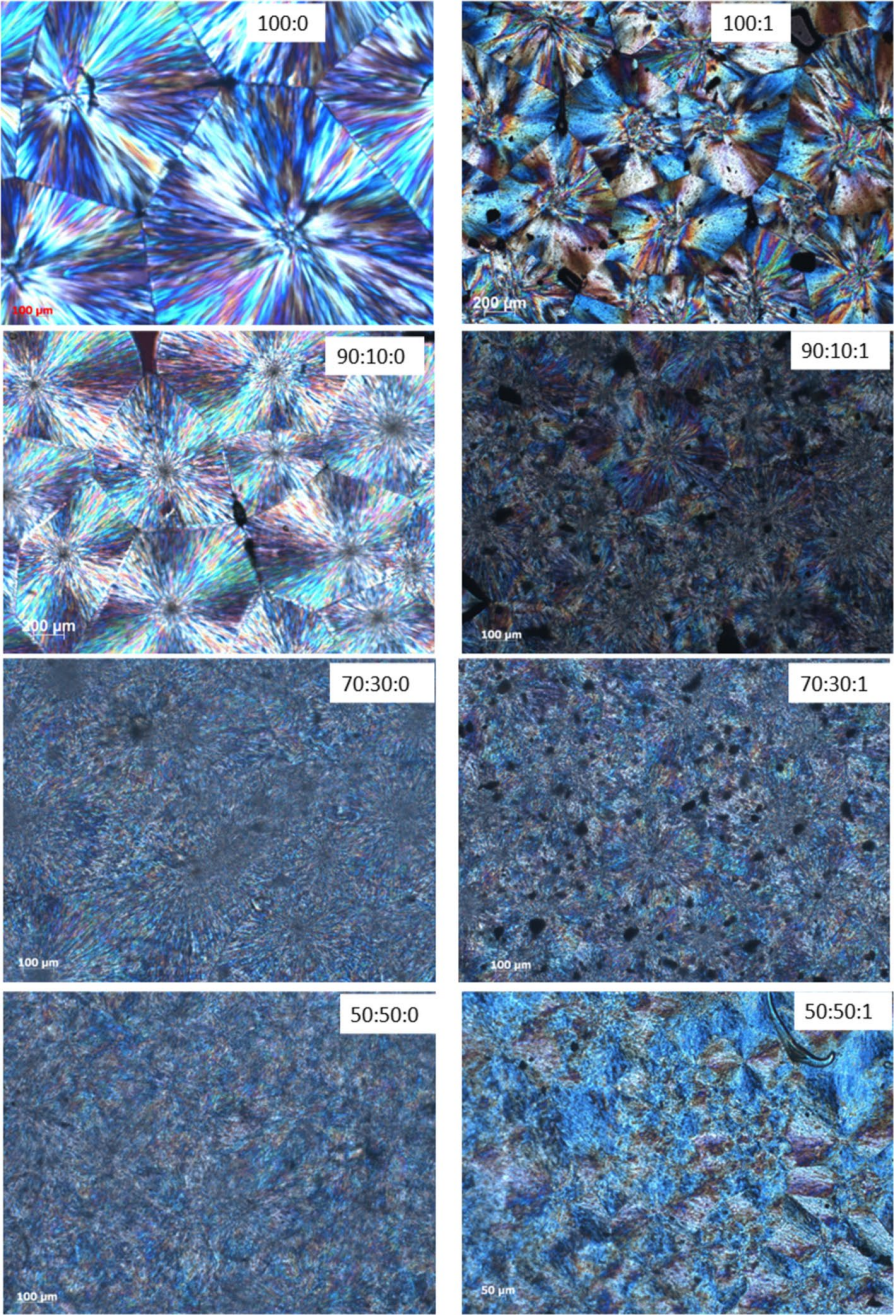
polarized optical micrograph for PLA: EVA80:TiO_2_ after isothermal crystallized at 125 °C for 90 min.

Figure 4 shows the size and morphology of PLA spherulites after isothermal crystallization at 125 °C for 90 min. When comparing the PLA/EVA 80 with the neat PLA, adding EVA 80 decreased spherulite size and increased their number. PLA's spherulitic structure and spherulitic boundaries in the blends are not as sharp as in the neat PLA. When the EVA80 content increases, it can be seen that the spherulite morphology changes significantly. This is because the copolymer enters intraspherulitic regions, causing the crystal superstructure to lose perfection (non-birefringent zones). This has been seen in several blends containing partially miscible or immiscible components^44^. The spherulite texture in blends with a lower EVA80 content (10 wt%) is almost identical to neat PLA. However, in higher EVA80 concentrations (30 wt% and 50 wt%), the texture mainly became irregular and open with diffused borders, showing that copolymer molecules were partially included into the amorphous zones between the crystalline lamellae of PLA and incorporated into the spherulites. The findings showed that EVA 80 inclusion could increase the nucleation density of PLA and a strong indication of compatibility between PLA and EVA 80. Therefore, one can expect an improvement in mechanical properties upon adding EVA80. Hoch et al. reported that the blown film of a blend of PLA with EVA 80 (80:20) exhibited better mechanical properties than that of immiscible PLA/EVA 60^45^. It was previously mentioned that TiO_2_ acted as a nucleation site for PLA^11,38^. In the case of PLA/EVA 80/1 wt% TiO_2_, increasing crystal nuclei results in smaller and denser spherulites.

**Figure 5:**
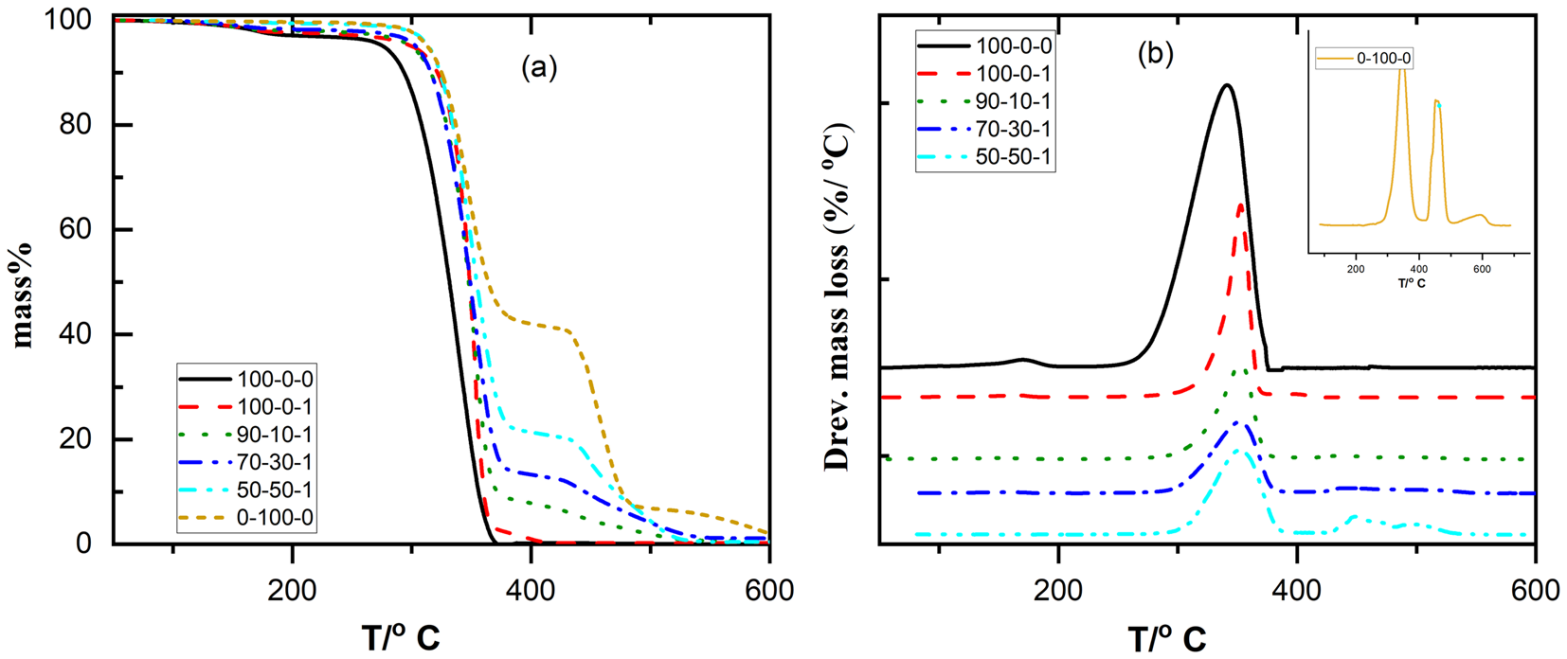
TGA (**a**) and Drev. Mass loss (**b**) curves of PLA: EVA80: 1 wt% TiO_2_ with a heating rate of 10 °C/min.

Figure 5 displays the TG and DTG thermograms of neat PLA, EVA 80, PLA/1 wt% TiO_2_ and PLA/EVA 80 loaded with 1 wt% TiO_2_. Neat PLA showed only one loss decomposition stage within the 300–350 °C temperature range ascribed to the chain scission of the PLA into several products, including lactides, cyclic oligomers, acetaldehydes, carbon monoxides, carbon dioxide, and acrylic acids^46,47^. As summarized in the data in Table 3, the onset decomposition (Tonset) of this loss was found to be at 267 °C with a temperature of maximum mass loss rate (Tp) at 341 °C. For PLA/nano-TiO_2_ thermal stability enhancement occurred, as evidenced by the increase of T_onset_ by 10 °C which agrees with the previous report^10^. Native EVA 80 exhibits two stages of decomposition; the first one started at 314 °C with a maximum of 346 due to deacetylation of the pendant acetate groups of EVA, while the second stage of decomposition with a maximum temperature at 461 is attributed to thermal scission of the backbone methylenic chain^48,49^. For PLA/ EVA80/ nano-TiO_2_ blends, the TGA profile exhibits two distinct stages of thermal decomposition. The initial mass loss observed between temperatures of 300–380 °C is attributed to the disintegration of the PLA component and the deacetylation of EVA. The second mass loss took place between 380 and 460 due to the decomposition of the main chain scission of the main backbone of the polyethylene and deacetylated EVA phases and releasing unsaturated butene and ethylene compounds in gaseous form. A comparison, based on the values of onset and maximum decomposition temperatures, was made between the thermograms of PLA/EVA80/TiO_2_ composites and neat PLA (Table 3), indicating that incorporating EVA80 and TiO_2_ nanoparticles improved the thermal stability of PLA. Previous reports have observed the same trend^7,12^.

**Table 3:** TGA results of PLA/EVA80/TiO_2_ composites.

Samples name	T_5%_ (°C)	T_50%_ (°C)	T_max_ (°C)	T_(90%)_ (°C)
PLA: EVA80:TiO_2_				
100:0:0	276	331	341	356
100:0:1	300	348	353	360
90:10:1	300	348	353	372
70:30:1	304	349	349	445
50:50:1	314	354	355	470
0:100:0	314	364	346 461	476

### Non-isothermal cold crystallization kinetics

**Figure 6:**
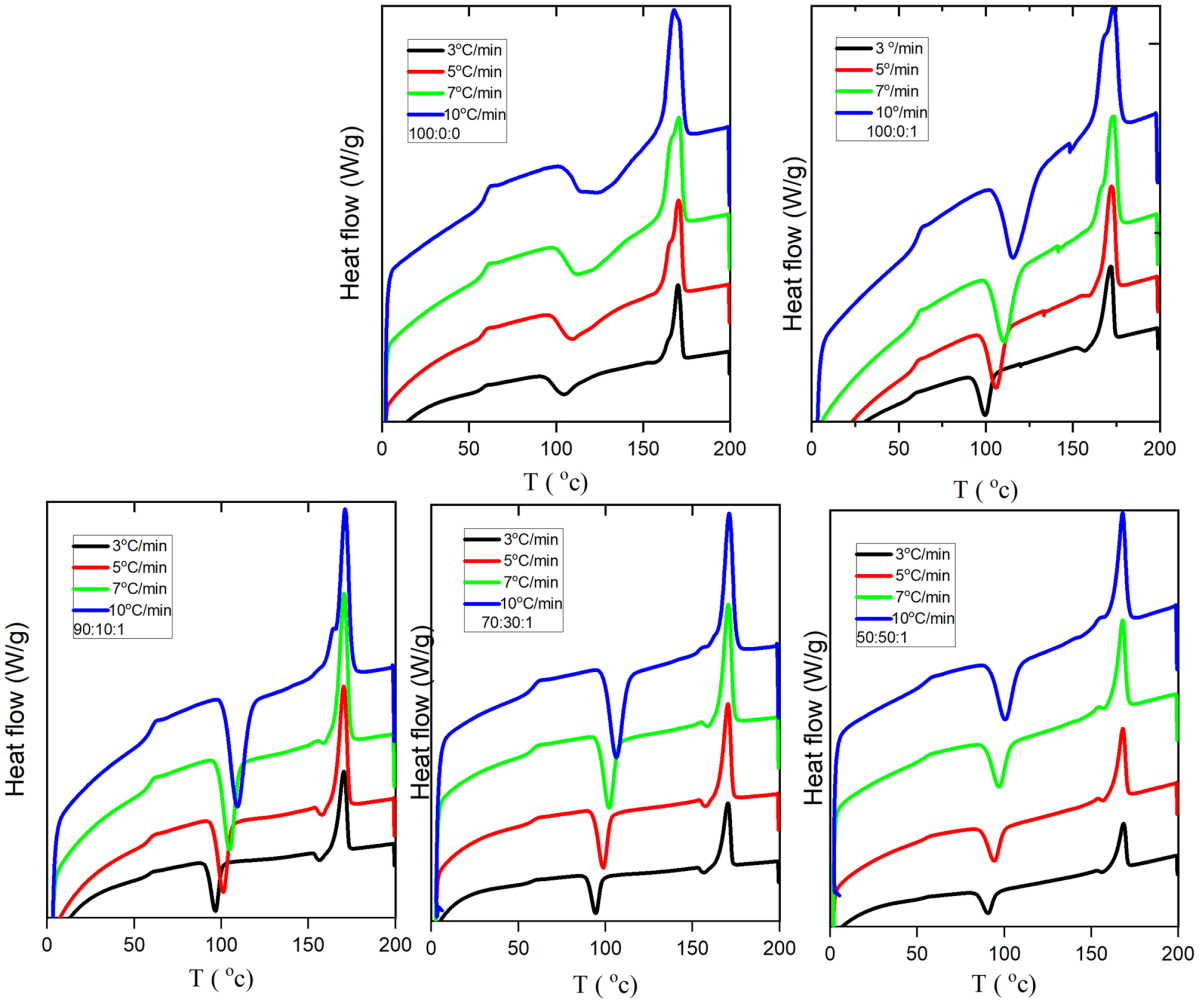
non-isothermal DSC different heating curves of PLA: EVA80: 1 wt% TiO_2_.

Figure 6 displays the DSC thermograms of neat PLA, PLA/1 wt% TiO_2_ and PLA/EVA 80/TiO_2_ for a second heating scan at different heating speeds. Exothermic cold crystallization peaks appeared in the curves at temperatures between 104 and 123 °C for neat PLA and 100–116 °C for PLA/1 wt% TiO_2_ and 95–106 for PLA/EVA80/1 wt% TiO_2_. When EVA80 or TiO_2_ are added to PLA, a distinct, sharper, narrower cold crystallization peak occurs during the heating scan. Figure 6 and Table 4 show that the cold crystallization temperature of neat PLA shifts to a lower temperature range as the heating rate is reduced. It demonstrated that increased crystallinity could be attained with lower heating rates (Table 4) and suggested that the lower heating rate enhanced crystallization by providing an appropriate crystallization time.

**Table 4:** Modified Avrami plot for PLA: EVA80:1 wt.% TiO_2_.

Name of PLA samples	$\varphi$ (°C/min)	${T}_{c}$ (°C)	$\Delta {H}_{c}$ (J/g)	xc	$n$	$log{Z}_{t}$	${Z}_{c}$× 10^−2^ (min^−1^)	${R}^{2}$	t_0.5_ (Min)	CRP × 10^−2^
PLA										
100:0	3	105	31	33	2.0	− 1.4606	32.59	0.999	4.5	4.7
	5	110	26	28	1.86	− 1.0228	62.43	0.999	3.0	
	7	113	25	27	1.92	− 0.8549	75.49	0.999	2.4	
	10	124	20	21	1.99	− 0.6085	86.92	0.999	1.8	
100:0:1	3	100	31	33	2.99	− 1.29	37.15	0.999	2.4	5
	5	106	33	35	2.85	− 0.8498	67.61	0.999	1.7	
	7	111	34	36	2.64	− 0.5745	82.77	0.999	1.5	
	10	116	38	40	2.42	− 0.4329	90.51	0.999	1.3	
PLA:EVS80: 1 wt% Tio_2_										
90:10:1	3	97	29	34	3.1	− 1.0794	43.67	0.999	2	8.4
	5	101	31	36	3.0	− 0.5019	79.36	0.999	1.3	
	7	105	34	40	2.9	− 0.2491	92.13	0.999	1.08	
	10	109	35	41	2.7	− 0.0419	99.04	0.999	0.9	
70:30:1	3	95	21	32	3.0	− 1.0385	45.06	0.999	1.9	9.4
	5	99	24	36	2.9	− 0.4583	80.97	0.999	1.3	
	7	102	25	38	2.9	− 0.1534	95.08	0.999	1.0	
	10	106	26	40	2.8	0.04883	101.13	0.999	0.85	
50:50:1	3	91	17	36	3	− 1.386	34.51	0.999	2.5	8.0
	5	95	17	36	3	− 0.803	69.07	0.999	1.6	
	7	97	18	38	3	− 0.535	83.84	0.999	1.3	
	10	101	19	39	2.8	− 0.181	95.90	0.999	1	

The same trend was also observed for PLA/EVA80/TiO_2_ blends. The cold crystallization enthalpy for neat PLA strongly depends on the heating rate. However, cold crystallization enthalpy for PLA/EVA 80/1 wt% TiO_2_ slightly depends on the heating scan, as shown in Table 3. The relative crystallinity X(T) as a function of crystallization temperature T would be obtained by integrating $\left(\frac{\partial H}{\partial t}\right)$ in the specified crystallization temperature range, as shown in Eq. (3).

2 $$\text{X}\left(\text{T}\right)= \frac{{\int }_{{T}_{0}}^{T}\left(\frac{\partial H}{\partial t}\right)\partial t}{{\int }_{{T}_{0}}^{{T}_{\infty }}\left(\frac{\partial H}{\partial t}\right)\partial t}$$

${T}_{0}$, $T$, and ${T}_{\infty }$ are the starting, arbitrary, and final crystallization temperatures. It is possible to translate the heating-induced crystallization temperatures to crystallization times based on Eq. (3)

3 $$t= \frac{\left|T-{T}_{0}\right|}{\varphi }$$

where $\varphi$ is the heating rate.

**Figure 7:**
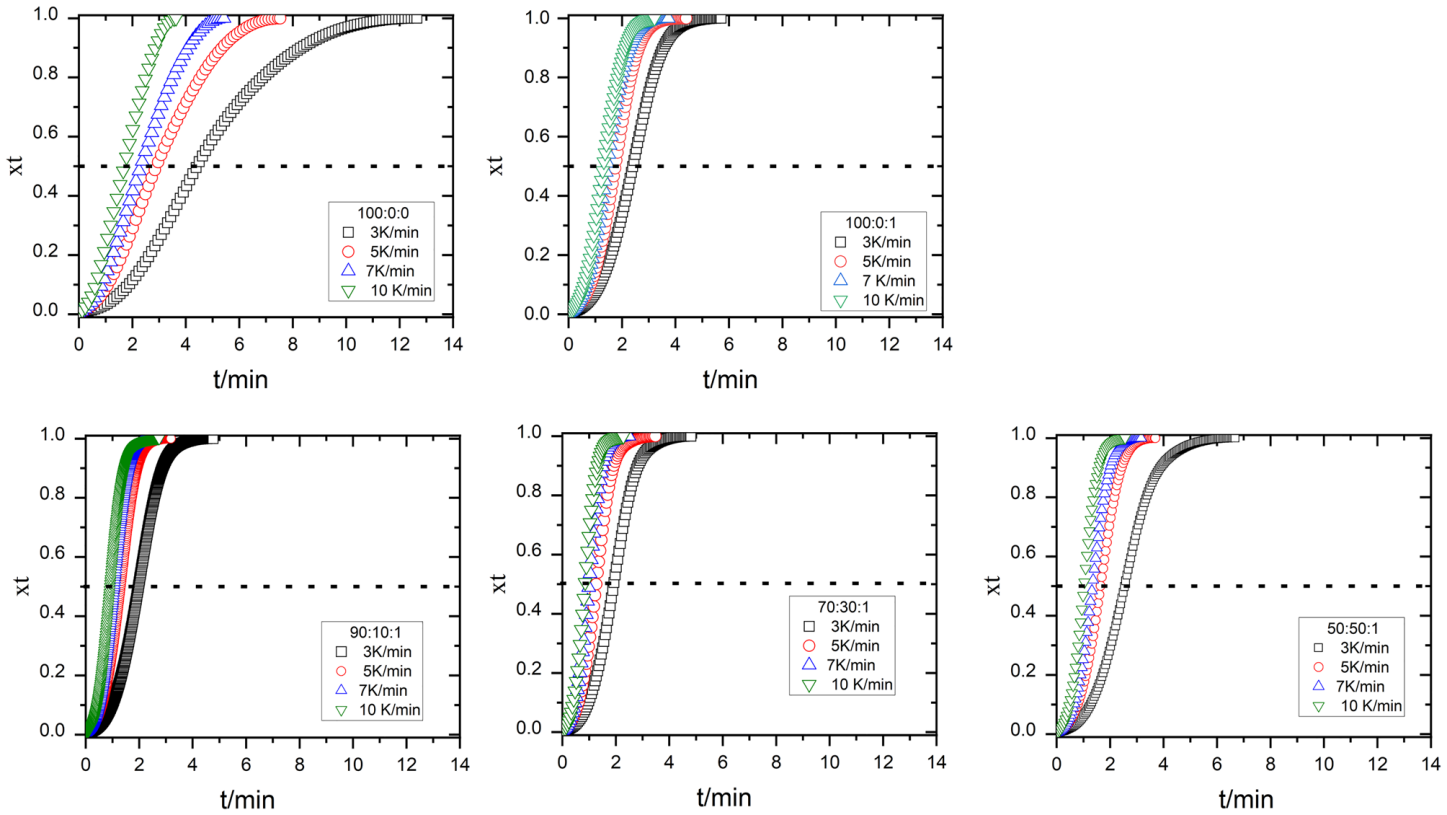
Relative crystallinity, $\text{X}\left(\text{t}\right)$ versus crystallization time for non-isothermally crystallized PLA: EVA80: 1 wt% TiO_2_ composites at different cooling rates, dot lines refer to t_0.5._

The relationship between the relative degree of crystallinity x(t) and the crystallization time t at different heating rates is shown in Fig. 7.

The time taken for 50% of total relative crystallinity is known as the crystallization half-time ($${t}_{0.5})$$. ($${t}_{0.5})$$ is evaluated from Fig. 7, and its value is inserted in Table 4. $${t}_{0.5}$$ is inversely proportional to the crystallization rate of polymers. Table 2 shows that the value of $${t}_{0.5}$$ decreases with the increase in the EVA80 content and the presence of TiO_2_ nanoparticles.

**Figure 8:**
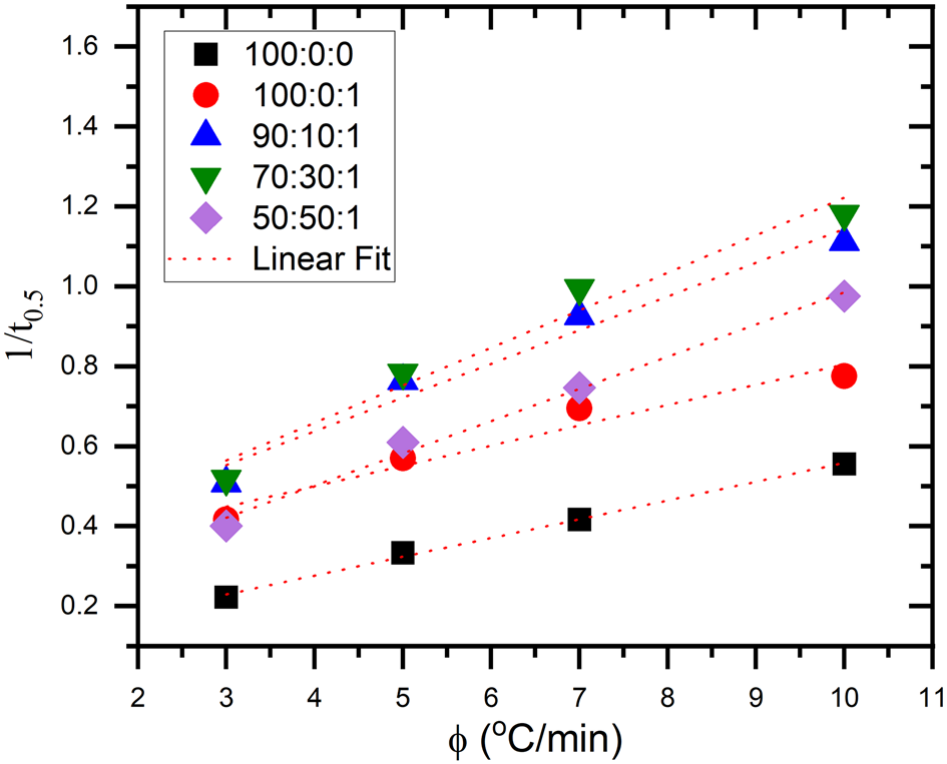
$\frac{1}{{\text{t}}_{0.5}}$ versus a heating rate for PLA:EVA80: 1 TiO_2_ composites.

The Crystallization Rate Parameter (CRP) is utilized to compare non-isothermal crystallization rates quantitatively. The assessment is conducted by analyzing the gradient of a linear graph created by plotting the inverse of ${(t}_{0.5})$ against the rate of heating, as seen in Fig. 8. Straight lines were achieved in this investigation for neat PLA and its composites, with a regression coefficient of 0.95. The value of CRP was evaluated and recorded in Table 4. A higher slope value suggests a more rapid rate of crystallization. The CRP value indicates that the rate of crystallization increases as the EVA 80 content increases. The correlation between CRP and EVA 80 levels indicates the compatibility between PLA and EVA 80.

It was reported that a completely immiscible blend is slightly CRP-dependent on blend composition^50^.

Jeziorny's modified Avrami equation^51^ can be employed to analyze the kinetics of non-isothermal crystallization in crystalline polymers^6,49,52,53^

4 $$\text{log}[-\text{ln}\left(1-X\left(t\right)\right]=\text{log}{Z}_{t}+n \,log(t)$$

where X(t) represents the relative crystallinity at time t. The Avrami exponent and crystallization rate constant are n and ${Z}_{t}$ respectively. Because temperature fluctuates constantly during non-isothermal crystallization, the constants Z and n have different physical meanings than isothermal crystallization. According to Jeziorny’s proposal, ${Z}_{t}$ needs to be modified by the heating rate as follows

5 $${Z}_{c}= \frac{\text{log}{Z}_{t}}{\varphi }$$

**Figure 9:**
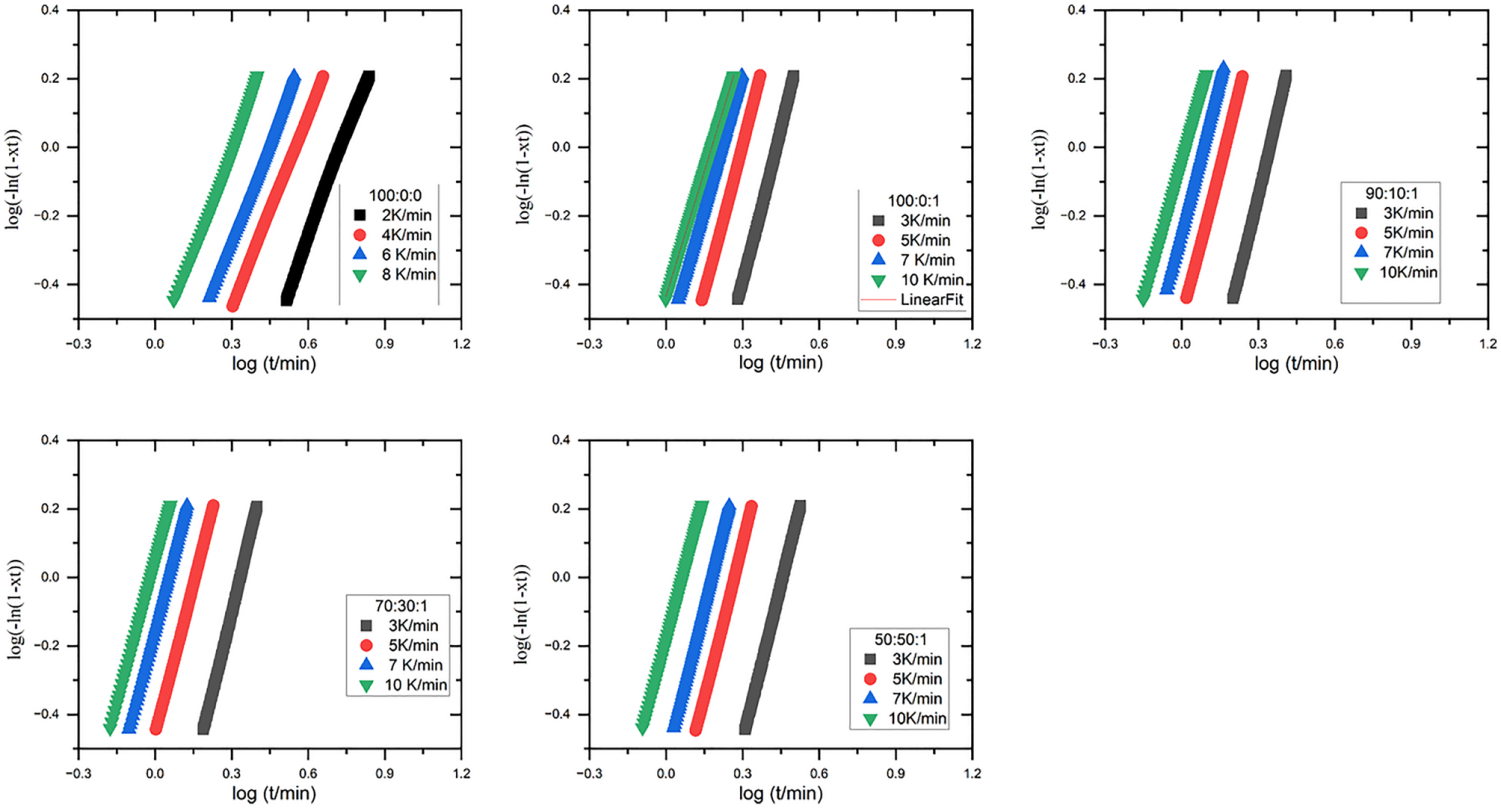
log(− ln(1 − xt)) versus log(t) for PLA: EVA 80: 1 wt% TiO_2_.

Figure 9 shows the modified Avrami plot for PLA samples for x(t) in the range of 0.2–0.8. For all PLA samples in Table 4, ${Z}_{c}$ rises as the heating rate increases, whereas t_1/2_ exhibits the reverse pattern. ${Z}_{c}$ is in the order: PLA/EVA 80/1 wt% TiO_2_ > PLA/1 wt% TiO_2_ > PLA. The typical Avrami exponent n values are 2.53 for pure PLA, 3.48 for PLA with 1 wt% TiO_2_ composite, and 3.7 for PLA/EVA80 with 1 wt% TiO_2_, indicating three-dimensional (3-D)^6,52,54^

The MO approach, which combines Avrami and Ozawa models, is frequently utilized in research to evaluate non-isothermal crystallization:

6 $$\text{ln}\left(\varnothing \right)=Ln(F\left(T)\right)- \alpha \text{ln}(t)$$

**Figure 10:**
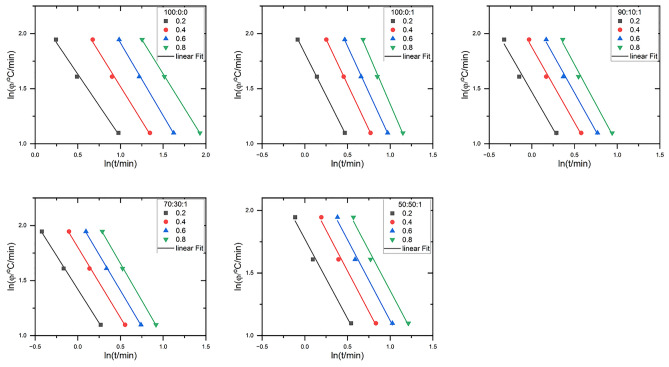
$\ln \left( \emptyset \right)$ versus $\text{ln}(\text{t})$ for different $\text{X}\left(\text{t}\right)$ of PLA: EVA 80: 1 wt% TiO_2_ composites.

Mo variables related to heating rate, $\varnothing$, and crystallization temperature. The plot of $\text{ln}(\varphi )$ against $ln (\text{t)}$ at a given relative crystallinity $X(t)$ is plotted in Fig. 10, where the slope and intercept of the curve are $\alpha$ and $\text{lnF(T)}$, respectively.

All data give a straight line from $\text{X}(\text{t})=0.2$–0.8, with a regression of 0.999 (as shown in Fig. 10; indicating that the MO approach could describe the crystallization behavior of the PLA and PLA/EVA 80/1 wt% Ti O_2_ composites well. A similar pattern was observed for PHB/EVA60^50^. Table 5 displays the values of “F(T)” and α for PLA and its composites. As a general trend, an increase in the degree of crystallinity shifts ${\text{the}}$ $\text{F(T)}$ values to higher values. PLA/EVA 80/TiO_2_ composites $\text{F(T)}$ values are lower than that of neat PLA. This suggests an enhanced effect of EVA80 and TiO_2_ on the crystallization behavior of PLA. However, when the concentration is considered at a given $\text{X(t)}$, the dependence of $\text{F(T)}$ values of PLA/EVA80/1 wt% TiO_2_ on concentration is weak due to slight miscibility between EVA 80 and PLA. Table 5 demonstrates the slope values. $\text{(b)}$ are almost constant for each composite's composition.

**Table 5:** MO approach parameters for PLA/EVA80/1 wt% TiO_2_ nanocomposites.

Sample	wt%	Kinetics parameter	$X(t)$			
			0.2	0.4	0.6	0.8
PLA: EVA80: TiO_2_	100:0:0	$F(T)$	9.02	15.8	24.5	33.1
		α	1.14	1.25	1.32	1.25
		${R}^{2}$	0.99	0.991	0.997	0.999
	100:0:1	$F(T)$	6.0	11.0	14.9	24.5
		α	1.59	1.63	1.68	1.80
		${R}^{2}$	0.999	0.999	0.999	0.98
	90:10:1	$F(T)$	4.5	6.7	9.0	11.0
		α	1.34	1.37	1.39	1.44
		${R}^{2}$	0.988	0.988	0.988	0.988
	70:30:1	$F(T)$	4.1	6.0	7.8	10.1
		α	1.23	1.28	1.30	1.34
		${R}^{2}$	0.98	0.98	0.99	0.99
	50:50:1	*F(T)*	5.8	8.67	11.13	14.15
		α	1.27	1.30	1.29	1.29
		*R*^*2*^	0.96	0.99	0.99	0.99

### An effective activation energy

By using Friedman's differential isoconversional approach, as described in Eq. (8), to the non-isothermal cold crystallization curve, it is possible to determine an effective activation energy ${E}_{X(t)}$ for PLA and its composites.

7 $${\left[\frac{\partial \mathit{ln}(X(t)/t)}{{\partial T}^{-1}}\right]}_{x(t)}= -\frac{{E}_{X\left(t\right)}}{R}$$

**Figure 11:**
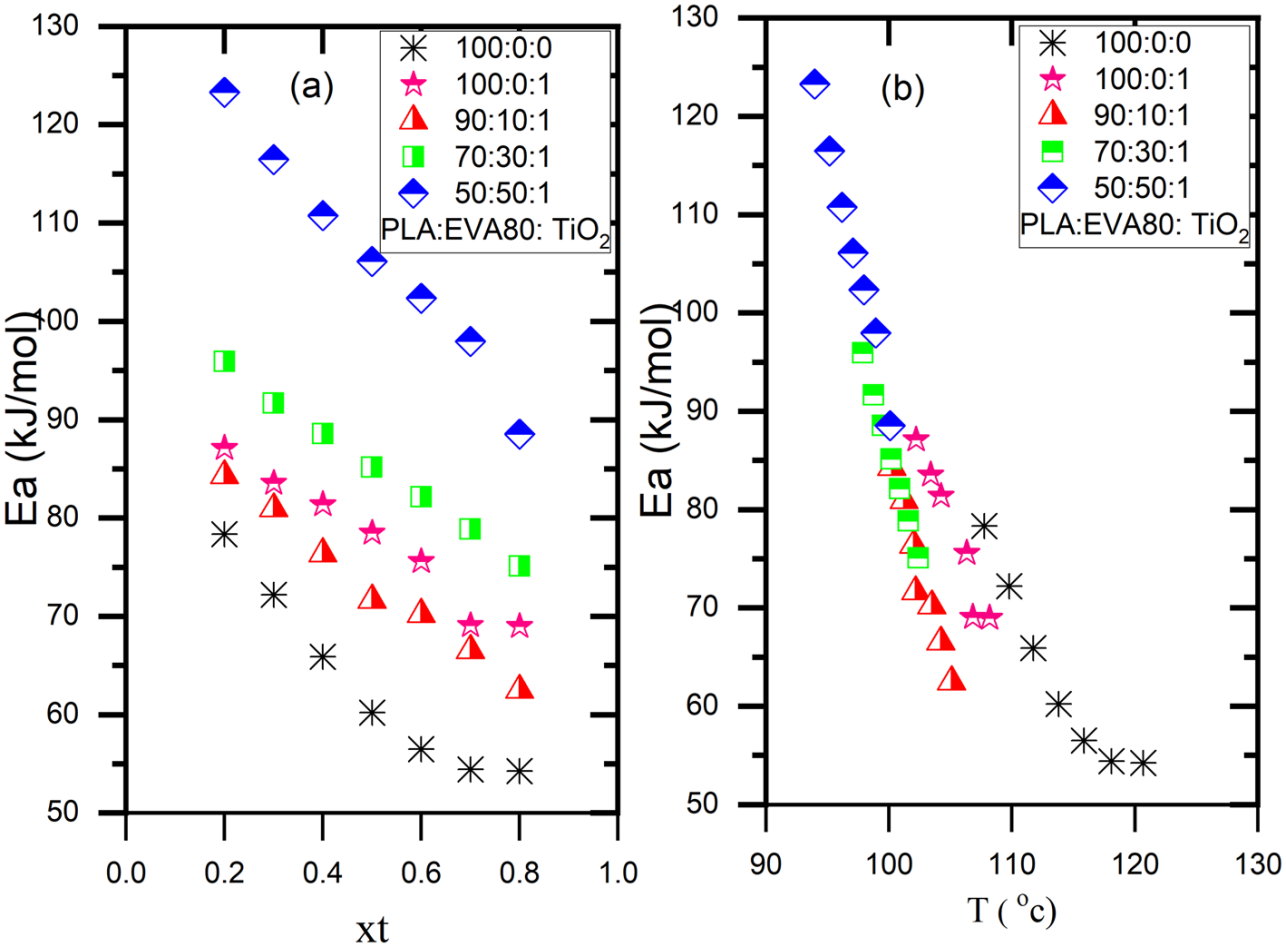
An effective activation energy as a function of relative crystallinity and average crystallization temperature.

$T$ is the temperature and $\mathit{ln}(X(t)/t)$ is the instantaneous crystallization for a certain relative crystallinity. For a given relative crystallinity, the slope of a linear plot of $\mathit{ln}(X(t)/t)$ as a function of 1/T is equal to ${E}_{X(t)}$, where ${E}_{X(t)}= -slope*R$. It is worth noting that the combined activation energy of the crystal growth and nucleation processes makes up effective activation energy. The effective activation energies of neat PLA and its composites at each relative crystallinity are positive, as seen in Fig. 11. This circumstance demonstrated that the crystallization rate increased as the crystallization temperature decreased^6,52,53^. Vyazovkin and Dranca et al.^55^found that a positive effective activation energy is obtained during non-isothermal crystallization when heating from the glassy state in the crystallization temperature zone above the glass transition temperature but below the region of maximum crystallization rate. Effective activation energy values dropped as relative crystallinity increased X(t), as seen in Fig. 11a. PLA/PEVA80/TiO_2_ exhibits higher effective activation energies than neat PLA. Reports^6,52^ have indicated a comparable pattern with PLA blends and composites. Figure 9b plot shows the relationship between effective activation energy and average temperature. The average temperature is computed at each X(t). Figure 11b shows crystallization occurred for PLA/EVA80/TiO_2_ nanocomposites at lower temperatures. This result is consistent with the DSC outcomes in Fig. 1 and Table 1. A similar trend has been reported for poly(butylene adipate-co-terephthalate)/treated calcium carbonate^54^, PLA/talc^56–58^, PLA/PHO/talc^6^

In future work, it’s important to explore different real processing techniques, such as 3D printing or molding, to study the effectiveness of these composites in practical applications.

## Conclusion

This study is the first attempt to examine, under nonisothermal crystallization circumstances, the effects of EVA 80 and nanosized TiO_2_ particles on the PLA's crystallization behavior starting from the glassy state. The Avrami and MO Approaches were successfully applied to evaluate the kinetic parameters of the PLA: EVA 80:1 wt% TiO_2_. The kinetic parameters, such as reaction rate (k), half-life time (t_0.5_), and MO parameter $F(T)$ as well as the effective activation energy (Ea), demonstrated that EVA 80 and 1 wt% TiO_2_ nanoparticles considerably accelerated PLA's overall crystallization compared to pure PLA. Furthermore, polarized optical micrographs reveal that the spherulitic structure of PLA significantly changed with the incorporation of EVA 80. Moreover, the nucleation density of PLA increased with the presence of EVA80 and TiO_2_ nanoparticles. SEM showed a random distribution of TiO_2_ nanoparticles. Moreover, no discrete domains of EVA 80 and many elongated fibrils indicate good adhesion between PLA and EVA 80. The depression of cold crystallization temperature and a slight lowering of the value of modulated glass transition temperature of the PLA phase may indicate the compatibility and good adhesion between PLA and EVA80. The presence of TiO_2_ nanoparticles and EVA 80 leads to improved thermal stability of PLA. From the above, one can expect that these unique combinations allow for a wide range of applications, from packaging to medical devices.

## Data Availability

The authors declare that the data supporting the findings of this study are available within the paper. Should any raw data files be needed in another format, they are available from the corresponding author upon reasonable request.
